# Head circumferences measured during developmental monitoring visits before diagnosis of childhood-onset craniopharyngioma

**DOI:** 10.1371/journal.pone.0307395

**Published:** 2024-07-23

**Authors:** Julia Beckhaus, Junxiang Peng, Svenja Boekhoff, Brigitte Bison, Carsten Friedrich, Hermann L. Müller

**Affiliations:** 1 Department of Pediatrics and Pediatric Hematology/Oncology, University Children’s Hospital, Carl von Ossietzky University of Oldenburg, Klinikum Oldenburg AöR, Oldenburg, Germany; 2 Department of Neurosurgery, Nanfang Hospital, Southern Medical University, Guangzhou, China; 3 Department of Neuroradiology, Diagnostic and Interventional Neuroradiology, Faculty of Medicine, University Hospital of Augsburg, Augsburg, Germany; PSTU: Patuakhali Science and Technology University, BANGLADESH

## Abstract

**Background:**

Craniopharyngiomas (CP) are histologically benign (WHO grade 1), embryonal malformations which are related to remnants of the Rathke’s pouch and are located in the (peri)sellar region. Already before CP diagnosis, many patients show a reduced growth velocity and tend to present with weight gain. However, it is unknown whether patients with CP develop an increased head circumference (HC) before CP diagnosis, which could be a useful early diagnostic indicator.

**Patients and methods:**

For a cohort of 83 patients recruited in the multicenter studies KRANIOPHARYNGEOM 2000 and HIT-ENDO data on HC could be analyzed, based on medical records assessed in developmental monitoring visits performed at defined time points before CP diagnosis.

**Results:**

When comparing HC standard deviation scores (SDS) before CP diagnosis in 83 patients at defined time points between birth and 4 years of age, all HC were in the upper normal range. However, CP patients diagnosed at an age ≤4 years with initial hypothalamic involvement presented with a tendency towards an increased HC SDS early before CP diagnosis at routine medical examinations during the first 7 months of life.

**Conclusions:**

We conclude that monitoring of growth and weight development including HC can lead to early CP diagnosis and treatment. This might prevent higher grades of hypothalamic involvement and lead to an improvement of quality of life after CP. Further studies on the specific value of HC as a diagnostic marker are warranted.

## Introduction

Craniopharyngiomas (CP) are benign embryonal malformations, which arise from ectoblastic remnants of Rathke’s pouch and can be found in hypothalamic and pituitary regions—both of importance in (neuro-)endocrine regulation and satiety modulation [1–3]. CP are the most common intracranial tumors of non-glial origin in the pediatric population, constituting between 6 to 9% of pediatric brain tumors. Overall there are 0.5 to 2 new cases of CP per million population occurring each year, 30 to 50% of which are diagnosed in children and adolescents [4]. The peak incidence is at an age 5 to 10 years but they can occur at any age, including infancy and pre- and neonatal periods [2]. Despite the tumor being benign (WHO grade I) [5] and a high overall survival rate of the patients, the quality of life is impaired and there is extensive morbidity [6–8]. Obesity as a manifestation of hypothalamic syndrome is present postoperatively in up to 52% of patients, with at least half of these patients having severe difficulty controlling their desire to eat [9–12].

Due to the rareness of the disease, it is important to increase the awareness for this complex diagnosis in general pediatric practice. Early diagnosis can contribute to a timely management of endocrine complications in CP patients, hence indicators for a punctual diagnosis are needed. Based on the embryonic origin, clinical manifestations and symptoms can frequently be observed early before CP diagnosis [13, 14]. Increased weight development occurred rather late in history approximately two years before CP diagnosis. Hypothalamic tumor involvement and a family history of obesity are risk factors for the development of severe obesity [15]. Clinical relevant impairments in height velocity have been observed in CP patients with hypothalamic involvement at a very young median age of 10–12 months [13].

Macrocephalus can be present as a family trait or e.g. in the context of Neurofibromatosis type 1 without any evidence for increased intracranial pressure. Increased intracranial pressure as a cause of macrocephalus can occur during early infancy most frequently due to inflammatory/infectious, oncological, bleeding-related diseases, and cerebral malformations. Sutures and fontanelles close during the first two years of life. However, early or delayed closures are signs of normal variants in healthy children or can also show an underlying pathology [16]. HC and the development of macrocephalus before diagnosis of CP has not been analyzed systematically in the context of this disease.

## Materials and methods

### Patient cohorts

This retrospective observational study used data collected in 90 patients recruited between December, 1^st^ 2000 and June 30^th^, 2007 in the multicenter studies KRANIOPHARYNGEOM 2000 (Clinical trial registration number: NCT01272622) and HIT-ENDO. This cohort of 90 patients was screened for medical records of their developmental monitoring visits from September 1^st^, 2017 till December 31^st^, 2017. After data collection, the data of individual participants were pseudonymized. The authors could not identify individual participants after data collection. The results of height and weight development before and after CP diagnosis in these patients have been published previously [13].

Data on HC (before CP diagnosis) was available in 83 of 90 patients (92%). Data was categorized by age at diagnosis to differentiate the groups that were diagnosed with CP during (≤4 years age at diagnosis) or after (>4 years age at diagnosis) their developmental monitoring visits. This cut-off point was mainly related to the age-range of data available in our health survey. We have data on HC before CP diagnosis during the first 4 years of life. Consequently, we decided to look at CP patients diagnosed during this early age period in comparison to patients who were diagnosed with CP not during this early age but later at an older age (>4 years of age). We speculated that an increased HC as an early clinical manifestation of CP could be expected in patients with an age ≤4 years at CP diagnosis.

The histological diagnosis of adamantinomatous CP was confirmed by neuropathological reference assessment in all cases.

### Anthropometric measurements

HC data were acquired from patient child health records, standardized and financed by German health authorities and conducted by local physicians and pediatricians. Health supervision visits are recommended at standardized ages: at the time of birth (U1), U3: 4- to 6-weeks-old, U4: 3- to 4-months-old, U5: 6- to 7-months-old, U6: 10- to 12-months-old, U7: 21- to 24-months-old, and U8: 3.5- to 4-years-old. HC was measured using a non-stretchable tape, wrapped around the widest possible circumference of the head, placed on the forehead above the eyebrows, above the ears and over the most prominent part of the back of the head by the local pediatrician. HC was expressed as SDS according to the references of Prader *et al*. [17].

### Neuroimaging

Hypothalamic involvement was assessed by intraoperative microscopic inspection and/or imaging. All pre- and postoperative, axial, coronal, and sagittal magnetic resonance imaging (MRI) were reviewed by a reference neuroradiologist (B.B.), blinded for clinical and surgical information, to assess preoperative hypothalamic involvement and the degree of surgical resection.

### Statistical methods

We compared patient’s characteristics of two age strata: patients diagnosed with CP at an age of ≤4 years versus patients diagnosed with CP at an age older than 4 years and analyzed their HC SDS measured at ages between 4–6 weeks until 4 years (U3–U8). Statistical analyses were performed using R software, version 4.2.1. Data are displayed as median (range) or frequency (percent). Differences in non-normally distributed continuous variables were assessed using Wilcoxon-rank-sum test; categorical variables were analyzed using Chi^2^ or Fisher’s Exact test (if at least one cell had an expected count of less than 5). We dichotomized the presence of hypothalamic involvement (yes/no). HC with and without hypothalamic involvement were compared graphically, in the two age groups. A two-sided p-value of ≤0.05 was considered as statistically noticeable.

### Statement of ethics

All procedures performed in our study were in accordance with the ethical standards of the institutional and/or national research committee and with the 1964 Helsinki declaration and its later amendments or comparable ethical standards. The studies KRANIOPHARYNGEOM 2000 (Clinical trial registration number: NCT00258453) and HIT-ENDO were approved by the local standing-committee on ethical practice of the Medizinische Fakultät, Julius-Maximilians-Universität Würzburg, Germany (140/99), and written parental and/or patient consent was obtained in all cases.

## Results and discussion

HC of 83 patients (38 male / 45 female) as measured at defined time points before diagnosis of childhood-onset CP were retrospectively analyzed based on patient records. Only patients with CP tracked with the standardized German health survey qualified for inclusion in this study, eliminating a sizable percentage of the 387 patients recruited in the German KRANIOPHARYNGEOM studies (5). In this cohort of 83 patients, CP was diagnosed at a median age 8.2 years (range: 1.7 – 20.5 years). The median follow-up period after CP diagnosis was 14.8 years (range: 2.2 – 35.5 years). Ten patients (12%) were diagnosed with CP at an age of ≤4 years. A gross total resection was achieved in 37 patients (45%) at first neurosurgical intervention. Fourty-four patients (53%) initially presented with a CP involving hypothalamic structures and 24 patients (29%) with hydrocephalus at the time of CP diagnosis.

No statistically noticeable differences were found between the two strata (age ≤4 years at CP diagnosis vs. age >4 years at CP diagnosis) regarding gender distribution, follow-up interval, hypothalamic involvement and hydrocephalus at the time of CP diagnosis (**Table 1**).

**Table 1:** Characteristics of patients with childhood-onset craniopharyngioma (CP) recruited in KRANIOPHARYNGEOM 2000 and HIT-ENDO and presenting at an age ≤4 years and at an age >4 years at the time of CP diagnosis. Continuous variables are presented as median [range]; categorical variables as n (%). P values display results of Fisher exact test or Wilcoxon rank sum test. n.a. = not available.

Patient characteristics	Total cohort	≤4 years at	>4 years at	p value
		CP diagnosis	CP diagnosis	
n	83	10	73	
Sex, male / female	38 (46) /45 (54)	6 (60) / 4 (40)	32 (44) / 41 (56)	0.50
Age at CP diagnosis, years	8.2	3.1	8.8	/
	[1.7–20.0]	[1.7–3.6]	[4.1–20.0]	
Follow-up interval, years	14.8	18.0	14.7	0.18
	[2.2–35.0]	[8.0–27.5]	[2.2–35.0]	
Hydrocephalus at CP diagnosis	24 (29)	3 (30)	21 (29)	>0.90
n.a. data on hydrocephalus	30 (36)	4 (40)	26 (36)	
Head circumference SDS [17] at birth	-1.30	-1.30	-1.30	0.75
	[-6.00–0.00]	[-5.50–0.70]	[-6.00–0.700]	
Head circumference SDS [17] at 3.5–4 years	0.40	0.10	0.40	0.49
	[-3.60–3.00]	[-3.60–1.10]	[-1.80–3.00]	
Hypothalamic involvement [18]	44 (53)	5 (50)	39 (53)	>0.90
Degree of surgical resection				>0.90
Gross-total resection	37 (45)	5 (50)	32 (44)	
Incomplete resection	41 (49)	5 (50)	36 (49)	
n.a. data on degree of resection	5 (6)	0 (0)	5 (7)	

**Fig 1:**
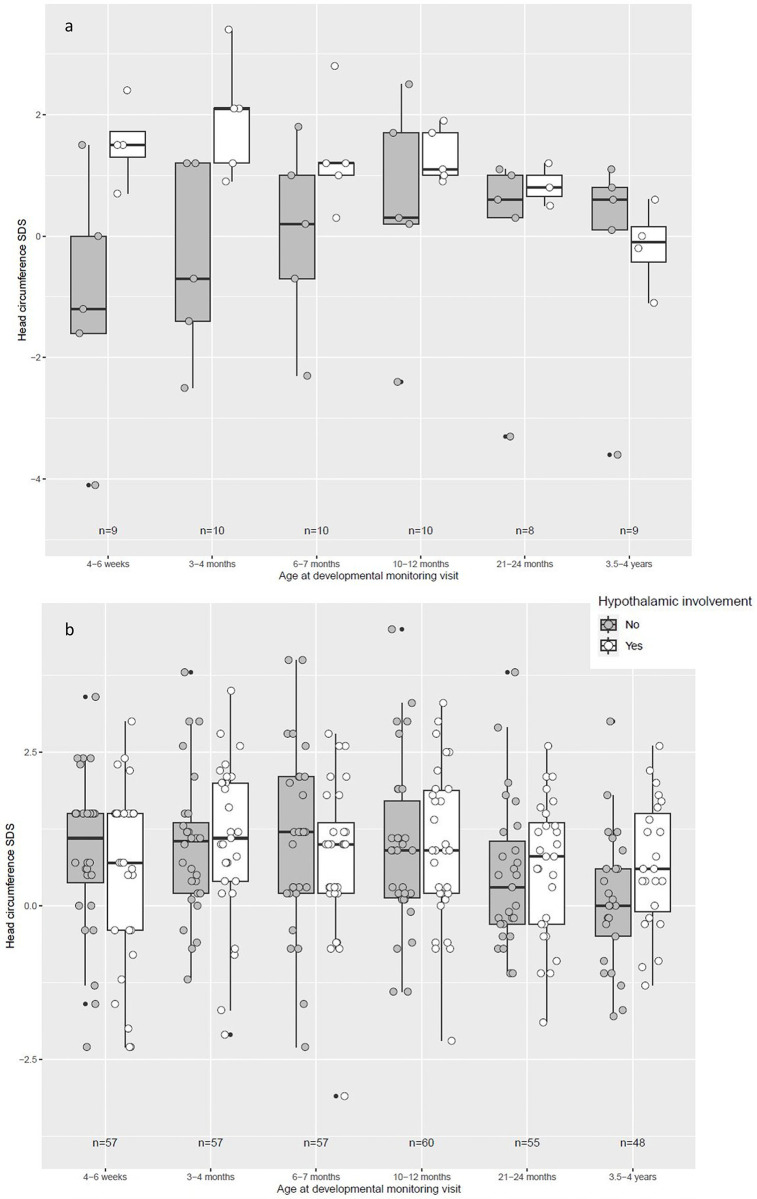
Head circumference standard deviation score (SDS) [17] in children diagnosed with childhood-onset, adamantinomatous craniopharyngioma (CP) and recruited in KRANIOPHARYNGEOM 2000 and HIT-Endo under or at (Fig 1A) and over (Fig 1B) the age of 4 years at the time of CP diagnosis. Data are shown as box plots for patients who presented with and without hypothalamic involvement (HI) of CP (open boxes = with HI; filled boxes = without HI). The horizontal line in the middle of the box depicts the median. The edges of the box mark the 25^th^ and 75^th^ percentiles. Whiskers indicate the range of values that fall within 1.5 box-lengths. Black bullets are depicting outliers. Individual measurements are indicated through circles.

Fifty-three percent (n = 44) of the patients presented with initial, hypothalamic involvement of the tumor at the time of CP diagnosis. Analysing HC in both groups with different age at CP diagnosis (≤ 4 years vs. >4 years of age at diagnosis), no statistically noticeable HC differences were detected with regard to hypothalamic involvement. When comparing HC SDS before CP diagnosis at defined time points between birth and 4 years of age, all HC were within the upper-normal range and no HC differences between patients with and without hypothalamic involvement of CP were observed (**Fig 1B**). In patients diagnosed with CP at an age of ≤4 years, a tendency was observed towards larger HC in CP patients with hypothalamic involvement indicating that hypothalamic involvement might be associated with a larger HC SDS at the examinations during the first 7 months of life (**Fig 1A**). However, analyzing the total cohort in terms of hypothalamic involvement and HC development, no statistically noticeable differences were found (S1 Table).

Childhood-onset CP is an embryonal, malformational tumor orginating and located in close proximity to the hypothalamic-pituitary axes (HPA) [7]. We have already reported on growth retardation as a very early symptom indicating CP with hypothalamic involvement at the end of the first year of life, i.e. several years before CP diagnosis [13]. As described in our previous study, body height and growth velocity decreased at an early age of 10–12 months and persisted until the diagnosis of CP. In the further development of the children from birth to an age of 5 years, a lower body height SDS was persistent over time. During the first 10 days of life, patients presented with a decreased BMI SDS. BMI increased 2–3 years before diagnosis of CP in patients at risk for morbid obesity i.e. in patients with hypothalamic involvement of CP. After surgical intervention, BMI SDS increased steadily during the first year after surgery. Patients with HI have shown a higher BMI SDS increase than patients without HI [13]. Hoffmann *et al*. [14] observed that the most frequent symptoms for CP noted in the patients’ records were headache, visual impairment, growth failure, weight gain and polydipsia or polyuria prior to CP diagnosis.

Accordingly, it can be speculated, that a congenital embryonal malformation such as childhood-onset CP located in the sellar/parasellar area with potential impact on cerebrospinal fluid (CSF) circulation out of the third ventricle might initially manifest with impaired CSF circulation, hydrocephalus, and increased HC early-on already before CP diagnosis [19]. Chentli *et al*. [20] reported on a CP case with a large HC at birth. The authors concluded that an enlarged HC can be used as an early indicator for CP diagnosis. The patient initially presented with blindness, hemiparesis and psychomotor retardation as further symptoms. Monitoring all signs of increased intracranial pressure is crucial to diagnose CP early and therefore to avoid life-threatening complications as neurological deficits or blindness [21].

On the basis of these speculations, hypotheses and case reports, we analyzed HC measured before CP diagnosis in CP patients recruited in our multicenter studies. Differences in terms of HC SDS between CP with or without hypothalamic involvement could not be observed in our present study. At all defined time points between birth and 4 years of age, HC of CP patients with hypothalamic involvement were within the upper-normal range (0 SDS to +4 SDS). However, in patients diagnosed with CP at an age of ≤4 years, a tendency was found that hypothalamic involvement might be associated with a higher HC SDS at the examinations during the first 7 months of life. Therefore, we speculate that a childhood-onset CP may be indicated not only by early and persistent reduction of growth velocity but also by an increase of HC during early infancy. It was noticeable that in the group, where no hypothalamic involvement was present, the HC SDS values were below the average and an increase within the first 7 months of life was present, showing a crossing of HC percentiles. Differential diagnoses with symptomatic increased HC could be e.g., traumatic perinatal, intracranial hemorrhage, acute or chronic inflammatory and infectious diseases, metabolic diseases, intracranial tumors, or idiopathic hydrocephalus. If there are, next to increased HC, endocrine deficiencies present, sellar masses involving the pituitary gland or treatment of sellar masses leading to surgical lesions of thy hypothalamic-pituitary axes can be suspected.

The results of our study are limited due to retrospective parts of the analysis and, as indicated, some observations are speculative at this point. To our knowledge, this is the first study examining HC in children with CP in a retrospective cohort study. Since the study was limited on our sample of 83 patients, further validations of the results are needed in larger cohorts. Nonetheless, a limitation of the study was the small sample size when we stratified for the age at diagnosis and hypothalamic involvement.

## Conclusions

We would like to conclude, that based on the results of our current study on HC and our previous studies [13] on the clinical relevance of height and weight development before CP diagnosis, the monitoring of growth rates is most important as a potential early symptom of CP with hypothalamic involvement detectable already in the age group of early infancy. In case of decreased growth velocity, further symptoms of CP such as polyuria/polydypsia, visual impairments, and also HC should be evaluated. In our craniopharyngioma registry, we will monitor HC of recruited CP patients also based on data of the health survey before CP diagnosis. To explore the prognostic potential of head circumferences, the results should be validated in an external and larger cohort. We hope that with larger samples and a comparison group associations between CP and HC could be clarified.

## Supporting information

S1 Table Head circumferences standard deviation score (SDS) [17] in children diagnosed with childhood-onset, adamantinomatous craniopharyngioma (CP) and recruited in KRANIOPHARYNGEOM 2000 and HIT-Endo with regard to hypothalamic involvement. (DOCX)
