# Supplementary material for: Head circumferences measured during developmental monitoring visits before diagnosis of childhood-onset craniopharyngioma
Source: PLoS One. 2024 Jul 23;19(7):e0307395. doi: 10.1371/journal.pone.0307395 (PMC11265653; doi:10.1371/journal.pone.0307395)
Supplement: S1 Table — (DOCX) [file pone.0307395.s001.docx]

**Supporting information**

**S1 Table. Head circumferences standard deviation score (SDS) [17] in children diagnosed with childhood-onset, adamantinomatous craniopharyngioma (CP) and recruited in KRANIOPHARYNGEOM 2000 and HIT-Endo** **with regard to hypothalamic involvement.**

|  | **Overall** | **No HI** | **HI** | **p-value** |
| --- | --- | --- | --- | --- |
|  | (N=83) | (N=39) | (N=44) |  |
| HC SDS at birth |  |  |  |  |
| Median [Min, Max] | -1.30 [-6.00, 0.700] | -1.40 [-6.00, 0.500] | -1.30 [-5.00, 0.700] | 0.56 |
| Missing | 12 (14.5%) | 5 (12.8%) | 7 (15.9%) |  |
| HC SDS at 2 weeks |  |  |  |  |
| Median [Min, Max] | -1.30 [-6.00, 0.700] | -1.30 [-6.00, 0.500] | -1.30 [-5.00, 0.700] | 0.72 |
| Missing | 14 (16.9%) | 4 (10.3%) | 10 (22.7%) |  |
| HC SDS at 4-6 weeks |  |  |  |  |
| Median [Min, Max] | 0.700 [-4.10, 3.40] | 0.700 [-4.10, 3.40] | 0.700 [-2.30, 3.00] | 0.91 |
| Missing | 17 (20.5%) | 6 (15.4%) | 11 (25.0%) |  |
| HC SDS at 3-4 months |  |  |  |  |
| Median [Min, Max] | 1.10 [-2.50, 3.80] | 1.00 [-2.50, 3.80] | 1.20 [-2.10, 3.50] | 0.12 |
| Missing | 16 (19.3%) | 6 (15.4%) | 10 (22.7%) |  |
| HC SDS at 6-7 months |  |  |  |  |
| Median [Min, Max] | 1.00 [-3.10, 4.00] | 1.10 [-2.30, 4.00] | 1.00 [-3.10, 2.80] | 0.88 |
| Missing | 16 (19.3%) | 5 (12.8%) | 11 (25.0%) |  |
| HC SDS at 10-12 months |  |  |  |  |
| Median [Min, Max] | 0.900 [-2.40, 4.50] | 0.900 [-2.40, 4.50] | 0.900 [-2.20, 3.30] | 0.61 |
| Missing | 13 (15.7%) | 4 (10.3%) | 9 (20.5%) |  |
| HC SDS at 21-24 months |  |  |  |  |
| Median [Min, Max] | 0.600 [-3.30, 3.80] | 0.400 [-3.30, 3.80] | 0.800 [-1.90, 2.60] | 0.29 |
| Missing | 20 (24.1%) | 7 (17.9%) | 13 (29.5%) |  |
| HC SDS at 3.5-4 years |  |  |  |  |
| Median [Min, Max] | 0.400 [-3.60, 3.00] | 0.100 [-3.60, 3.00] | 0.400 [-1.30, 2.60] | 0.18 |
| Missing | 26 (31.3%) | 9 (23.1%) | 17 (38.6%) |  |
